# Admittance Method for Estimating Local Field Potentials Generated in a Multi-Scale Neuron Model of the Hippocampus

**DOI:** 10.3389/fncom.2020.00072

**Published:** 2020-08-04

**Authors:** Clayton S. Bingham, Javad Paknahad, Christopher B. C. Girard, Kyle Loizos, Jean-Marie C. Bouteiller, Dong Song, Gianluca Lazzi, Theodore W. Berger

**Affiliations:** ^1^Department of Biomedical Engineering, Case Western Reserve University, Cleveland, OH, United States; ^2^Department of Electrical Engineering, University of Southern California, Los Angeles, CA, United States; ^3^Department of Biomedical Engineering, University of Southern California, Los Angeles, CA, United States

**Keywords:** neural network, finite-element (FE), local field potential (LFP), multi-scale, numerical algorithm, volume conduction, extracellular current measurements

## Abstract

Significant progress has been made toward model-based prediction of neral tissue activation in response to extracellular electrical stimulation, but challenges remain in the accurate and efficient estimation of distributed local field potentials (LFP). Analytical methods of estimating electric fields are a first-order approximation that may be suitable for model validation, but they are computationally expensive and cannot accurately capture boundary conditions in heterogeneous tissue. While there are many appropriate numerical methods of solving electric fields in neural tissue models, there isn't an established standard for mesh geometry nor a well-known rule for handling any mismatch in spatial resolution. Moreover, the challenge of misalignment between current sources and mesh nodes in a finite-element or resistor-network method volume conduction model needs to be further investigated. Therefore, using a previously published and validated multi-scale model of the hippocampus, the authors have formulated an algorithm for LFP estimation, and by extension, bidirectional communication between discretized and numerically solved volume conduction models and biologically detailed neural circuit models constructed in NEURON. Development of this algorithm required that we assess meshes of (i) unstructured tetrahedral and grid-based hexahedral geometries as well as (ii) differing approaches for managing the spatial misalignment of current sources and mesh nodes. The resulting algorithm is validated through the comparison of Admittance Method predicted evoked potentials with analytically estimated LFPs. Establishing this method is a critical step toward closed-loop integration of volume conductor and NEURON models that could lead to substantial improvement of the predictive power of multi-scale stimulation models of cortical tissue. These models may be used to deepen our understanding of hippocampal pathologies and the identification of efficacious electroceutical treatments.

## Introduction

Encouraged by advances in computation, new multi-scale neural network models have demonstrated the ability to predict spatiotemporal patterns of activity in complex neural tissue systems when activated by detailed models of stimulating electrodes (Cline et al., 2015; Gilbert et al., 2015; Bingham et al., 2016, 2018a,b). Linking neuronal and tissue volume-conductor scales has previously been performed by estimating interactions between extracellular electric fields induced by stimulating impulses and neural processes which lie within affected volumes. These tissue models conduct electricity both passively and actively yet do so only in a feedforward manner. However, neural tissue systems behave simultaneously as a volume conductor and as a network of spiking neurons with these two domains of tissue interacting bidirectionally. Active conductance through neural networks, occurring through changes in ion concentrations across cell membranes, results in measurable currents in the extracellular space that may entrain or reinforce oscillatory behavior in neural networks (Whittington et al., 1997; Fries, 2005; Anastassiou and Koch, 2015). These measurements, called local field potentials (LFPs), are critical indicators of living tissue system behavior. Despite the sophistication of feedforward modeling approaches, there remain doubts regarding the best method of calculating LFPs. Analytical methods, which have a strong theoretical basis, have been used successfully for many years (Clark and Plonsey, 1968, 1970; Holt and Koch, 1999; Lindén et al., 2011, 2014). However, analytical field estimation methods fail to capture the heterogeneous nature of neural tissue. For very complex and highly segmented volumes, both heterogeneous resistivity and anisotropy have been demonstrated to create important boundary conditions during tissue volume conduction (Nowak and Bullier, 1996; Grill, 1999; McIntyre and Grill, 1999; Bossetti et al., 2008; McIntyre, 2009; Miocinovic et al., 2009; Bazhenov et al., 2011; Joucla and Yvert, 2012; Howell and McIntyre, 2016). Thus, numerical approaches, including the finite-element and finite-volume methods, have become favored over analytical methods for two reasons: numerical methods allow (i) more efficient parallel/simultaneous estimation of multiple locations within an electric field and (ii) superior ability to capture various dielectric heterogeneities of complex nervous tissue systems. Therefore, it is warranted that numerical methods be extended to incorporate transmembrane currents into dynamic estimates of extracellular electric fields generated within complex neural tissue systems.

Simulation of neural tissue systems, through large-scale computational modeling, has become possible through dramatic improvements in computation over the past decade, namely: parallel computation through either graphic-processor or more traditional CPU cluster computing. When combined with numerical estimations of electromagnetic fields, computational models of neurons have demonstrated utility as a method of optimizing neurostimulating or recording arrays of electrodes (Geddes, 1997; Johnson and McIntyre, 2008; Rattay et al., 2012; Agudelo-Torom and Neef, 2013; Howell and Grill, 2014; Howell et al., 2015; Bingham et al., 2018a; Buccino et al., 2019). With respect to LFP estimation, numerical methods have already been used with some success, however no thorough analysis of the theoretical foundations of the approach has been performed (Fernández-Ruiz et al., 2013). Despite advancements in these modeling approaches, there remain significant limitations in numerical methodologies for incorporation of endogenous currents into estimations of local field potentials (LFP) or evoked potentials (EP) for use in analyzing the stimulus-responses of neurological tissue systems. Barriers to be overcome, include proving the unestablished theoretical protocols for tetrahedral or hexahedral approximation of a line-source or point-source within a conductive volume and designing a feasible process for tackling the great computational burden presented by the task. Such a theoretical solution would ideally allow currents to give rise to roughly toroidal or spherical electric fields generated by line or point-sources, respectively, in an infinite homogeneous conductive volume. But it is unclear at which length scales preserving these hypothetical field geometries matters for the accuracy of field estimations.

The Admittance Method (AM) has been established as a valid and intuitive numerical approach to estimating electric fields induced in animal tissue systems through extracellular electrical stimulation (Cela, 2010; Xie et al., 2011). The AM is a specialized form of the FEM method. In short, AM involves construction and solution of an equivalent RC circuit used to approximate the attenuation of electric fields in volume conductors. This paper seeks to distinguish the AM as a numerical approach to electromagnetics modeling that is capable of smoothly incorporating endogenous neuronal current sources into an inherited meshed model for prediction of evoked potentials in complex neural tissue systems. The proposed extension of this method yields the following utilities: (i) a granular algorithm and guidance for adding dynamic current sources to existing meshed volumes where spatial alignment between nodes and new current sources is variable, (ii) automated incorporation of very large numbers of dynamic current sources to existing AM meshed volumes, (iii) intuitive implementation of tissue volume conduction behaviors via equivalent circuits, (iv) simultaneous solution of electric fields at many locations, and (v) the ability to add dielectric heterogeneities to LFP estimation models. Overcoming these challenges allows us to take advantage of the computational efficiency and the potential for dielectric detail of numerical methods for solving volume conduction problems.

## Methods

The algorithms proposed herein were developed through utilization of data previously published and generated from a multi-scale model of a rat dentate gyrus. The following sections briefly summarize the construction, feedforward simulation, and validation of a complex multi-scale AM-NEURON model of the hippocampus (Bingham et al., 2016, 2018a). While this model is extensively described in previous publications, it benefits the reader for some concise explanations to be provided here.

### Construction of the NEURON Model of the Hippocampus

The rat dentate slice NEURON model was an *in-situ* scale and density computational reconstruction of histology comprising the Kjonigsen and Witter hippocampal atlas (Kjonigsen et al., 2011; Bingham et al., 2016, 2018a,b). Taking a particularly clear image from the atlas, the cell layer boundaries were extruded 400 μm in the septotemporal direction to yield the entire volume within which the model was constructed.

The NEURON model is comprised of 50,000 granule cells and 10,000 entorhinal cortical axon fibers. Previously validated granule cell models, including morphology and biophysics, were taken from Hendrickson et al. and arranged along the cell body layer of the image from the atlas (Hines and Carnevale, 2003; Hendrickson et al., 2016). Then 5,500 lateral perforant path axons were arranged such that they coursed through the outer third of the granule cell arbors and 4,500 medial perforant path fibers likewise through the middle third. Each granule cell then formed synapses with these fibers, prioritizing proximity, totaling ≈5.5 million synapses in the middle third and ≈6 million in the outer third.

### AM-NEURON Feedforward Simulation

Volume conduction in neural tissue in this study was modeled using a variant of the heterogeneous AM, as extensively described in Cela (2010), Xie et al. (2011), Bingham et al. (2018a). In brief, this method involves four steps: (i) image-based sculpting of a model volume and automated extraction of bounding surfaces between sub-volumes of differing conductivity, (ii) construction of a resistor (and capacitor) network of varying resolution which discretizes the defined volume, (iii) assignment of resistances to individual elements in the mesh based on experimentally measured resistivity (the estimated real component of impedance), and (iv) solution by conjugate-gradient descent of a sparse matrix representation of internode admittance and currents, and formulating nodal voltages according to Ohm's law (Zhang et al., 2006). This established method provides meaningful flexibility in terms of mesh construction, dielectric properties, and expression of sources within the resulting model when compared to common alternatives such as FEM via the COMSOL modeling environment (Al-Humaidi, 2001).

Because both neuronal and volume conductor models are electrical circuits, interfacing the two intuitively involves passing node voltages or currents back and forth (**Figure 2**). Once nodal voltages in the AM model are solved, voltage at neuronal compartments are found by tri-linear interpolation from the 8 nearest nodes and applying this voltage as an extracellular battery via the extracellular mechanism in NEURON—this is done for each compartment in every neuron (~200–300 locations for each neuron and about 12.25 million locations in an *in situ* scale slice of the dentate gyrus)–this causes currents within each cell that are appropriate given their location within the field estimated by the AM model.

Once the Volume conductor and network models were in place, we added additional circuit elements to represent virtual stimulating devices. The devices we modeled had real geometry and conductive properties of twisted bipolar platinum microwires insulated with Teflon. This was implemented with equivalent circuits by discretizing the tissue-electrode interface with parallel RC components, each representing a mode of current transmission: faradaic and capacitive charge injection (Cole and Cole, 1941; Geddes, 1997).

After this step, we placed the devices at different transverse locations and delivered a biphasic pulse at threshold amplitude. Compartmental currents from the infrapyramidal perforant path stimulation case (1 ms pulse-width per phase with no interphase delay at 150 μA) were then used to calibrate the AM for LFP estimation. This case was selected for LFP estimation analyses that follow because stimulating in the infrapyramidal blade allowed stimulation artifacts to be as far from the boundaries of the volume conductor model as possible, somewhat reducing the required complexity. This proved important owing to the dozens of variations of meshed models (complexity and geometry) that were used to analyze numerical vs. analytical performance.

The model was simulated on a 4,040-processor high-performance computing cluster owned by the authors and supported through the University of Southern California Center for High-Performance Computing.

### Analytical LFP Estimates as a Proxy Ground Truth

**Figure 1:**
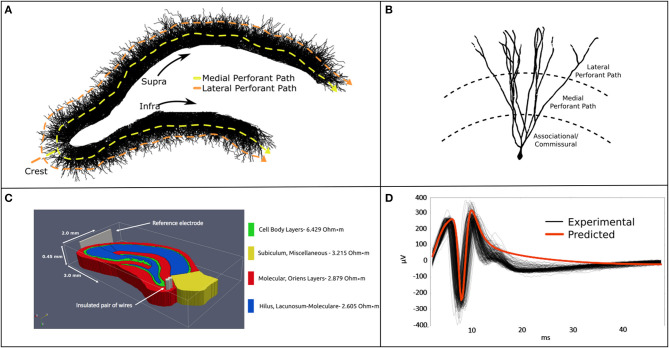
**(A)** A 2-D rendering of the biologically realistic NEURON model of a rodent dentate gyrus with demarcation of recording and stimulating devices. **(B)** The slice model is populated with 10 k entorhinal cortical axons which synapse with 50 k granule cells. The entire model comprises ≈12.25 million synapses and ≈15 million neuronal compartments. **(C)** The NEURON model was imbedded in a meshed volume with bipolar stimulating electrodes targeting the perforant pathway. **(D)** Analytical estimates of LFPs corresponded well with *in vitro* MEA recordings in temporal dynamics and waveform. Model validation was performed for stimulation at the crest perforant path and recordings from the suprapyramidal cell body layer. Details regarding the construction and application of this model are described at length in Bingham et al. (2018a).

Previous studies, by these authors and others in the community, used simple analytical estimations of local field potentials to validate their neuronal models (Figure 1). While the sources of error associated with such an approach are well-understood, the point-source method of LFP estimation provides reasonably accurate estimates of potentials at a given observation point in the model. By assuming conductive homogeneity (homogeneous, isotropic, and infinite boundary volume conductor), this estimate can be used as a suitable proxy for ground-truth when calibrating more sophisticated numerical approaches to estimating electric fields under the same conductive conditions (Einevoll et al., 2013). These assumptions reduce sources of error to numerical volume conductor implementation rather than the countless known and unknown factors influencing the emergence of extracellular signals in real neural tissue systems. Lastly, a homogeneous analytical method yields a level of experimental control and access to system variables that cannot yet be obtained by *in vitro* or *in vivo* electrophysiology. It is on this basis that we justify establishing a homogeneous analytical estimate of LFPs as a proxy ground-truth to validate the numerical methods developed in the sections that follow.

The analytical method used in this study is the point-source equation, as follows:

(1) $$\begin{array}{l} \phi \left(x,y,z\right)=\;\frac{1}{4\pi }\overset{n}{\underset{i=0}{\sum }}\frac{I_{i}}{\sigma^{*}r_{i}} \end{array}$$

(2) $$\begin{array}{l} r_{i}\;=\;\sqrt{{\left({x-x}_{i}\right)}^{2}+{\left(y-y_{i}\right)}^{2}+{\left({z-z}_{i}\right)}^{2}} \end{array}$$

Where ϕ is the field potential resulting from *n* current sources, *I*. σ is the inverse of tissue resistivity (3.8 Ω-m, obtained from Lopez-Aguado et al.) and r is the path length from source to recording location (Clark and Plonsey, 1970; Holt and Koch, 1999; López-Aguado et al., 2001; Wilson et al., 2014). The proxy ground-truth is estimated by scaling each current source via Equations 1 and 2, then summing them into a field potential using the principle of superposition. This process was performed for every current source and time step in a simulation until the whole time-series is reconstructed. LFP estimates using Equations 1 and 2 based on the feedforward AM-NEURON model described in this and previous paragraphs were validated against LFP recordings from MEA studies of rat hippocampal slices and published in Bingham et al. and Soussou et al. (Hentall et al., 1984; Soussou et al., 2006; Bingham et al., 2018a). The following methods describe a new analysis of this data.

### New Meshes for LFP Estimation: Tetrahedral vs. Hexahedral

**Figure 2:**
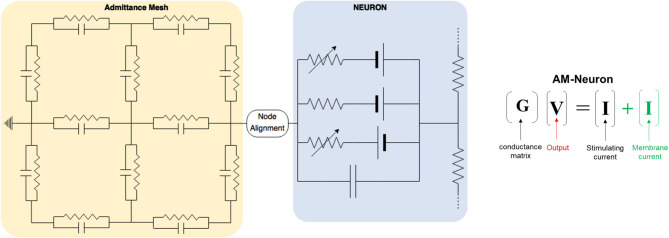
This toy model demonstrates the chief architecture of the equivalent circuits used to estimate the passive and active propagation of neural network activity in an AM-NEURON model. Volume conduction is modeled via a specialized resistor and capacitor network called the Admittance Method (yellow). Intracellular and transmembrane currents are then modeled using a second set of equivalent circuits setup and solved within the NEURON simulation environment (blue) (Hines and Carnevale, 2003). Differences in spatial resolution between these two domains draws attention to the impact of potential approaches to handling their interface (i.e., node alignment).

Early in this process it was observed that the spatial distribution of currents contributing to LFP signals differed substantially from the geometry of stimulating currents (i.e., many disperse small charges vs. a few large and nearly co-local charges) (Figure 2). Therefore, we determined to start with a fresh and less constrained set of meshes with which to study LFP estimation. Suppressing any preconceived notions of which mesh volume geometry would be best, a bounding box was fit to the point cloud that represented all compartments in the neuron model which would be contributing currents to any LFP estimate (**Figure 4**). A spatial scaling factor was then applied to expand this volume, ensuring that current sources near the edges of the model would be less affected by boundary-driven current shunting. The bounding volume scalar factor used in this study was 142%, which was set after determining that the relative field amplitude of edge-most currents are >95% relaxed by the time they reach the nearest boundary. This volume was then meshed with either grid-based hexahedral or unstructured tetrahedral (equifacial ideal) meshes with resolution (maximum edge-length) ranging from 40 to 180 μm edge-lengths. Tetrahedral meshes were constructed through Python scripts that make API calls to Gmsh 4.5 and TetGen 1.5.1 using Delaunay triangulation (Chew, 1989; Geuzaine and Remacle, 2009; Si, 2015).

### Fundamentals of Resistor Network Construction

**Figure 3:**
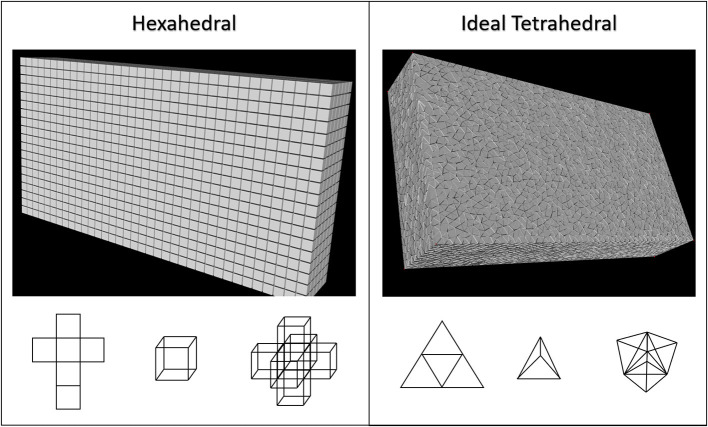
Two common mesh geometries, grid-based/structured hexahedral and unstructured tetrahedral were used to fill 3-dimentional volumes to encapsulate all current source locations described in this figure. The accuracy and computational performance of these were analyzed as mesh resolution was gradually increased in both. Hexahedral meshes were created by linear segmentation of the volumes to ensure voxels were always cubical. Tetrahedral meshes were scripted in python through the Gmsh and TetGen APIs using Delaunay triangulation to ensure very high quality (Geuzaine and Remacle, 2009; Si, 2015). It should be noted that tetrahedral meshes created using this approach do not have uniformly equifacial tetrahedra.

Tetrahedralizations or hexahedralizations (Figure 3) are unbroken networks of nodes and edges that must then be converted to a valid circuit of virtual resistors (and capacitors for non-quasistatic models). Rules for deriving mesh element resistances from experimental impedance or resistivity measurements are poorly described or completely absent from the literature, so we will describe our process here: first for hexahedral and then for tetrahedral mesh geometry. Because of the orthonormality of structured hexahedral meshes we assumed edge length, *L*, and edge cross-sectional area, *A*, to be the only scalars of importance in converting local tissue resistivity, ρ, to resistance, *R*, according to:

(3) $$\begin{array}{l} R=\rho \frac{L}{A} \end{array}$$

*A* was a constant 0.25 μm^2^ for all meshes in this study. Because unstructured meshes are not orthonormal, it stands to reason that there should be different rules for assigning or scaling distance-derived resistances based on the angle and number of adjoining connections. Following analysis done by Duffin in the late 1950's, fractional conductance within and across tetrahedra can be approximated as proportional to the cotangent of internal angles (Duffin, 1959), after the form:

(4) $$\begin{array}{l} R_{AB}=\frac{6\times \rho \times \tan\left(\vartheta \right)}{\left|CD\right|} \end{array}$$

**Figure 4:**
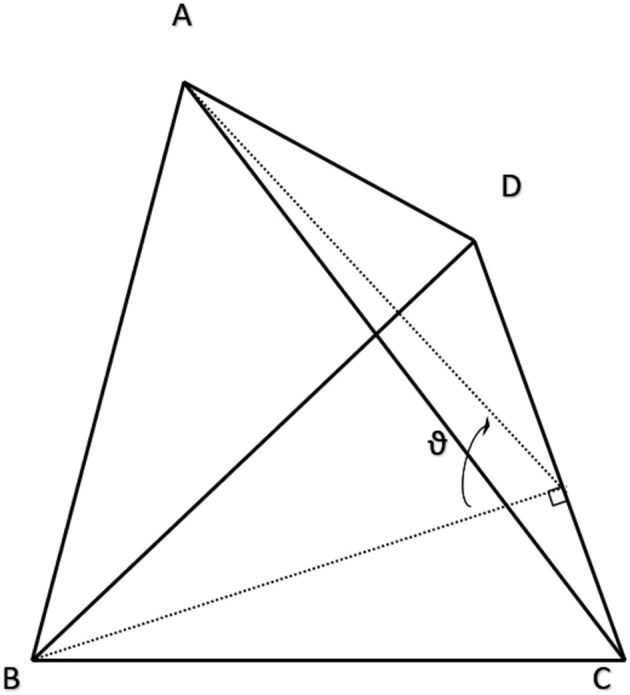
Example tetrahedral element explaining the variables used to apply the cotangent rule for calculating edge-wise conductance. Resistance from A to B is calculated by Equation (3) where *R*_*AB*_ is proportional to the ratio of the dihedral angle, ϑ, and the edge length, CD. A proof of this approach is presented at length in (Duffin, 1959).

Where resistance along an edge, *R*_*AB*_, across from the dihedral angle, ϑ, is 6 times resistivity times the tangent of ϑ scaled by the length of the edge where the dihedral angle is formed (variables described by Figure 4). The dihedral angle, ϑ, can be reliably calculated with the following equations after ensuring that u, v and w have the same sign:

(5) $$\begin{array}{l} \vartheta =\cot^{-1}\left(\frac{\sqrt{\left(u.u\right)}\left(v.w\right)-\left(u.v\right)\left(u.w\right)/\sqrt{\left(u.u\right)}}{u.\left(v.w\right)}\right) \end{array}$$

(6) $$\begin{array}{l} u=B-A,v=C-A,w=D-A \end{array}$$

The denominator in Equations 5 is the triple product of *u, v*, and *w*. This treatment of the tetrahedral meshing problem later requires that *R* be summed for edges shared by multiple conjoined elements to create a unified and continuous network without superfluous co-parallel resistors. This is done by following fundamental circuit-theory logic for summing parallel resistors:

(7) $$\begin{array}{l} \frac{1}{R_{Total}}=\frac{1}{R_{1}}+\frac{1}{R_{2}}+\ldots +\frac{1}{R_{n}} \end{array}$$

This overall approach of applying material properties to tetrahedral meshes (Equations 4–7) has the nice feature of *tangent*(ϑ) going to 0 as ϑ approaches 0 and infinity as ϑ approaches π/2. Therefore, very narrow elements are effectively short-circuited and meshes of a regular grid that yield tri-rectangular tetrahedra are electrically equivalent to hexahedralizations of the same grid. Whether this rule remains useful for obtuse tetrahedra was not studied, as none were generated in these models.

Following construction of a primary mesh and conversion to a circuit, new resistors were added between the hull nodes and a virtual ground. Each new wire connected to ground from a hull node had a resistance equal to the average of all other wires connected to the hull node being considered.

### Incorporating Incident Endogenous Current Sources: Splitting vs. Shifting

**Figure 5:**
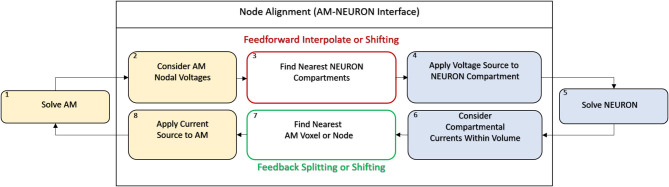
AM-NEURON bidirectional interface can be reduced to an intuitive and methodologically consistent algorithm that ensures field geometry distortion is low and mostly dependent upon mesh resolution at or near current sources. Eventually, this algorithm would be executed at each time-step in a simulation: first the Admittance Method model is solved, then the estimated field is applied to compartments throughout the NEURON model. Then, a single time-step of the NEURON model is solved and compartmental currents are passed back out to the Admittance mesh and the whole process begins again to prepare to simulate the next time-step. Spatial alignment can be accomplished with one of two algorithms: interpolating/splitting to/from a source or shifting a compartment or source to the nearest node in the AM mesh. The details of these two approaches are graphically explained in Figure 6.

**Figure 6:**
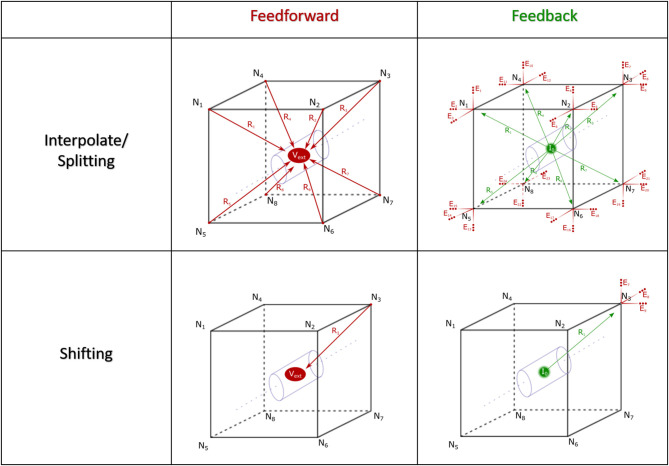
Spatial alignment in feedforward (AM to NEURON) and feedback (NEURON to AM) interfacing can be accomplished with one of two algorithms: interpolating/splitting to/from a source or shifting a compartment or source to the nearest node in the AM mesh. The accuracy and computational performance of solving meshes with split vs. shift were analyzed across a range of model complexities.

The fundamental paradigm of the AM was then extended to incorporate incident and dynamic current sources from NEURON compartments into an already existing meshed model (Figure 5). For the sake of clarity, a single voxel/single incident current-source scenario is presented in Figure 6. This figure describes two algorithms that will be labeled descriptively as, current “splitting” (i.e., interpolation) and “shifting.”

The steps for current splitting include, first, relative distance-weighted division of currents into eight or four parts, for hexahedral and tetrahedral elements, according to each of the nodes in the voxel that contains the source in the AM mesh. Secondly, these divided currents are then applied as a new static current source at each node.

This relatively complex algorithm was also compared with a simpler current shifting algorithm which applies all current sources at the nearest respective node in the mesh according to Figure 6 (shifting). This method became interesting because of the relative complexity of finding a voxel which contains a source location vs. just finding the nearest mesh node to that current source.

LFP estimations via these two algorithms were compared to each other and a point-source estimated LFP for both computational performance and estimate accuracy. Comparison was performed through calculation of a time-averaged absolute residual, or error, with respect to the analytical solution at recording locations evenly distributed throughout the transverse plane of the tissue model.

**Figure 7:**
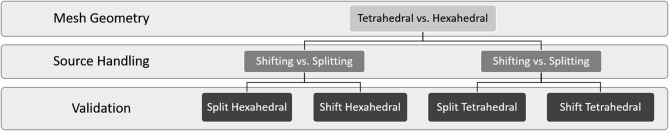
This flow diagram provides an overview of the analysis performed in this manuscript. To establish best practices for interfacing volume conduction models with large-scale NEURON network models, we explored the impact of mesh geometry and current source handling algorithms on computational performance and estimation accuracy. Validation consists of comparison of numerical model estimates with a proxy ground truth in the form of analytical estimates (line-source equation).

Once a resistor network was constructed and properly grounded, either the splitting or shifting algorithms were used to add a set of currents to the model. While the geometry of the model remained the same for every time step, a new netlist was written for each time step to account for changing current source amplitudes. Current source-node assignments remained the same for every time step for a given mesh geometry and resolution. These netlists were then loaded in series and nodal voltages were solved using the matrix solvers described at length by Cela (2010). Figure 7 provides an outline for all the analysis performed to compare the mesh geometries and current source handling algorithms described in this report.

### Model Sharing and Software Distribution

It is our eventual goal to release a software tool that will enable other investigators to optimize and later estimate LFPs within tissue models of arbitrary geometry and conductivity. Therefore, subsequent to acceptance of this manuscript, code to generate a meshed volume, integrate current sources, solve nodal voltages, and visualize results will be distributed via a public Github repository (https://github.com/bingsome/Neurospice). There remain, however, significant hurdles for adaptation of this method to different tissue systems, including but not limited to: tissue geometry, heterogeneous dielectric properties, and the complexities of constructing a biologically realistic NEURON model capable of generating meaningful virtual extracellular currents. Further, the value of such a tool may be somewhat reduced should we not focus future efforts on solving possible computational bottlenecks to be raised in the discussion portion of this manuscript.

## Results

The following sections describe results of a comprehensive study of the AM for LFP estimation. More specifically the outcome of sensitivity analysis surrounding mesh geometry (tetrahedral vs. hexahedral) and current source handling algorithms (splitting vs. shifting). Models were cross-compared for both computational performance and accuracy with error calculated with respect to point-source LFP estimates as a proxy ground truth. These estimates were made at recording locations evenly distributed throughout the transverse plane of the tissue model.

### Analytical LFP Estimates as a Proxy Ground Truth

**Figure 8:**
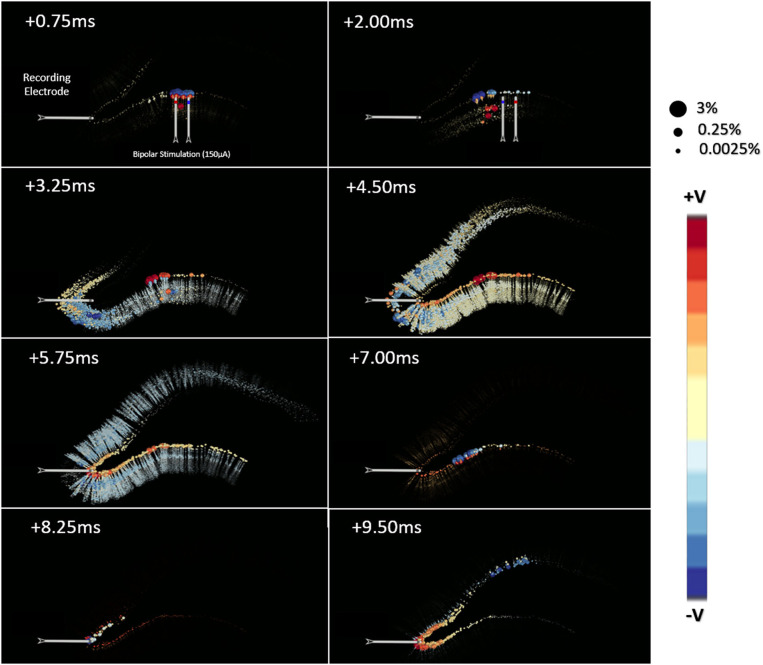
This visualization presents the source data, derived from simulations of a multi-scale model of an *in vitro* hippocampal slice, which was used to develop the models presented in this manuscript. The hippocampal model is extensively described in Bingham et al. (2018a). Each ball represents the location of a current source generated by a neuronal compartment in a hippocampal network simulation of perforant path stimulation. The color and size of each ball encodes the charge polarity and the normalized amplitude of the voltage each ball contributes to the potential as measured at the recording electrode. This normalization was performed for each time-step independently using the line-source equation (Holt and Koch, 1999). A feature of note in this data set is that the spatial distribution of important current sources varies dramatically from beginning to end of the simulated behavior.

The point-source equation was used to estimate LFPs in 1222 evenly distributed locations on a 2d-grid within a transverse plane at 50 μm under the stimulating electrodes and centered over the NEURON model. Figure 8 shows the impact that these neuronal current sources have on a single LFP recording taken near the crest. The calculations used to create Figure 8 were repeated to estimate the analytical LFP at all 1222 locations in a grid distributed throughout the transverse plane of the tissue model. This process was performed according to Equations 1 & 2 with a tissue resistivity of 3.8 Ω-m along all possible paths in an infinite boundary volume conductor. Completion of this estimate took ≈25 min for a single recording location but ≈18 h for 1222 locations due to some multi-threaded parallel optimization that was possible. While analytical method estimation speed could plausibly be further optimized, it was not the goal of this study to improve analytical estimation but rather to validate numerical modeling approaches.

### Incorporating Incident Endogenous Current Sources: Split vs. Shift

**Figure 9:**
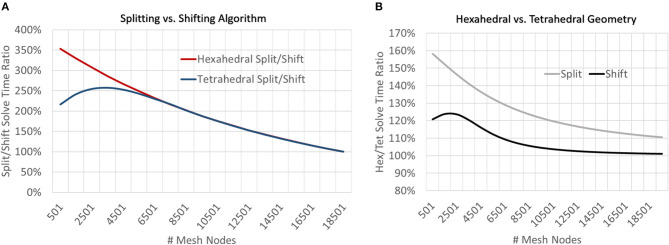
To examine the impact of current source handling algorithms (splitting vs. shifting) and mesh geometry (tetrahedral vs. hexahedral), meshes of a range of resolutions were solved and solve times were compared by both geometry and current source handling algorithm. **(A)** At low resolutions, shifting sources outperformed splitting for both mesh geometries but the relative performance converged to parity at greater model resolutions (≈19 k nodes). **(B)** For all mesh resolutions studied, tetrahedral meshes were solved more quickly than hexahedral, though this difference was diminished when using the shifting current handling algorithm and performance parity was achieved at higher model resolutions (≈15 k mesh nodes).

**Figure 10:**
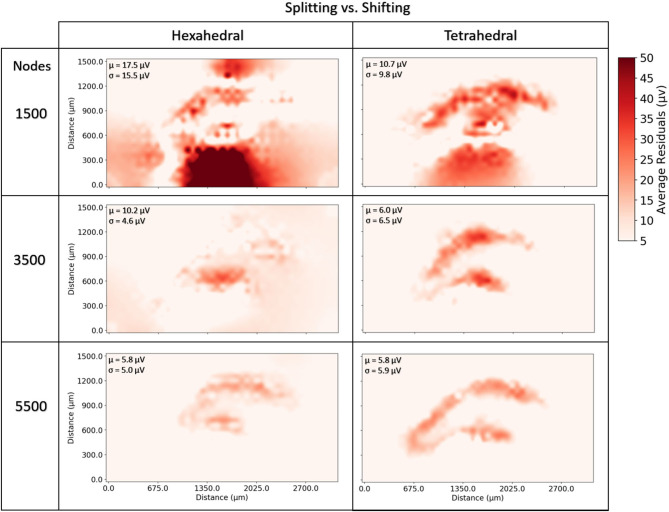
To examine the estimation accuracy of numerical LFP solutions under differing current source handling methodologies (shifting vs. splitting) a range of models with varying resolution were generated, solved, and quantitatively compared with an analytical estimate via the line-source equation in the plane at 50 μm under the slice model, and then with each other. Red regions represent voxels where shifting results in more error relative to splitting. μ, σ in each window denote the average and standard deviation of residual differences over all voxels and all time-steps. While splitting often outperformed shifting, particularly in tetrahedral meshes at very low resolutions, as meshes became more complex, shifting, and splitting methods reached comparable levels of error (low single digit μV). The difference in methods across the range of resolutions was more pronounced in hexahedral meshes for any given node density because of the relative element size between hexahedral and tetrahedral meshes.

At very coarse resolutions current splitting had the advantage of 11–30% better accuracy at a computational penalty of 220–350% longer solve times (Figures 9, 10). These coarse model differences were more exaggerated for hexahedral meshes due to the greater number of nodes per mesh element. Regardless of mesh geometry, differences in accuracy quickly dissolved (low single digit μV on average) by the time node counts reached ≈5–6 k (Figure 10).

Because the number and spatial distribution of current sources remained the same for every meshed model, the preprocessing step of assigning sources to voxels or nodes was particularly impactful for very coarse meshed models. While this step involves pairwise relational distance calculation for millions of points and, therefore, increases in complexity with increasingly complex meshes, preprocessing time did not increase nearly as fast as did the time required to solve the circuits.

### New Meshes for LFP Estimation: Tetrahedral vs. Hexahedral

**Figure 11:**
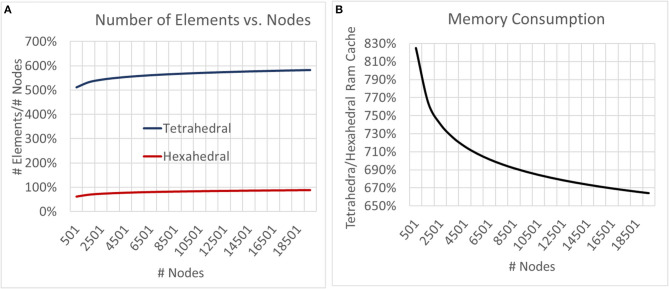
To examine the impact of geometry on model and computational complexity we created meshes of unstructured tetrahedral and structured hexahedral geometry across a range of spatial resolutions and compared the **(A)** number of elements required to mesh a set of nodes and **(B)** the relative RAM utilization of the generated models. As models grew in complexity the tetrahedral to hexahedral element to node ratio converged to ≈6x with tetrahedral models utilizing ≈6.5x more memory.

**Figure 12:**
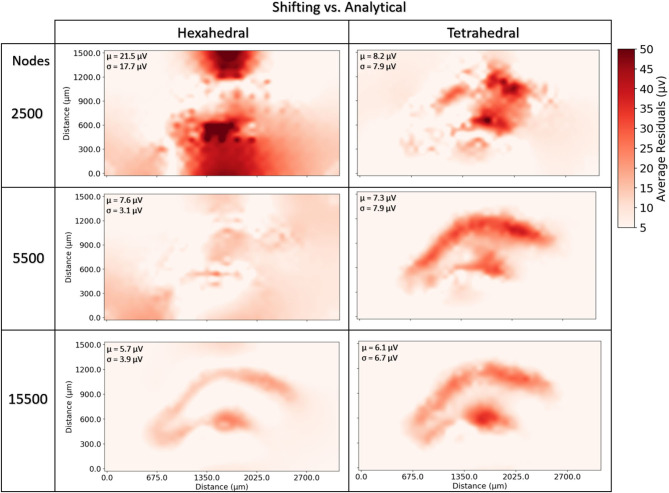
To examine the estimation accuracy of numerical LFP solutions under differing mesh geometries (tetrahedral vs. hexahedral) and managing endogenous current sources via the current shifting regime, a range of models with varying resolution were generated, solved, and quantitatively compared with an analytical estimate via the line-source equation in a plane at 50 μm under the slice model. μ, σ in each window denote the average and standard deviation of residuals over all voxels and all time-steps. Hexahedral meshes achieved accuracies comparable to tetrahedral meshes at around 1/6th the number of elements. Also, hexahedral solutions at higher node counts achieved a smoother solution (visible in the bottom row and in σ's).

Neither tetrahedral nor hexahedral meshes were universally superior in the analyses performed. For very coarse meshes, tetrahedral models were markedly faster to solve and could be more accurate than hexahedral but not without substantial cost in both dynamic memory utilization and model complexity (Figures 9–12). As models increased in resolution, speed and accuracy differences between the two geometries diminished but tetrahedral models maintained ≈6–6.5x greater complexity and memory requirements due to higher element to node ratios. At very high resolutions, hexahedral meshes yielded solutions that were slightly more accurate on average but significantly smoother than tetrahedral solutions with a similar mesh node-count.

### Sample LFP Estimates From the Medial Molecular Layer

**Figure 13:**
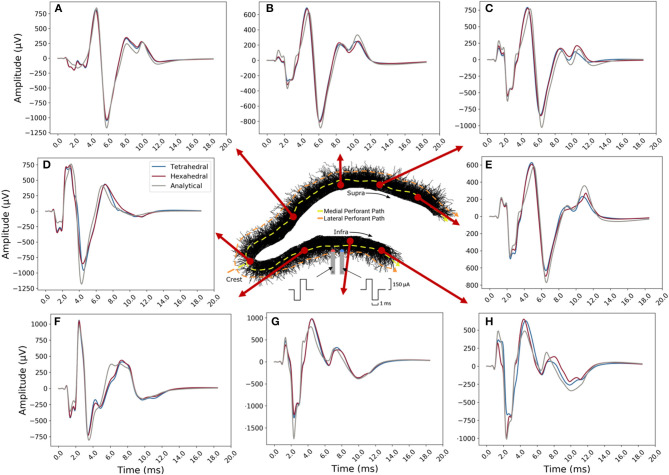
Example LFP time-series for current shifted tetrahedral (blue) and hexahedral (red) models plotted alongside the analytical (line-source equation; gray). Solutions were calculated along the transverse axis with **(A–E)** along with enclosed or suprapyramidal blade, **(D)** at the crest, and **(F–H)** along the exposed or infrapyramidal blade of the dentate gyrus. The tetrahedral and the hexahedral meshed solutions each had 6,500 nodes. Predictions were best in the suprapyramidal blade (top), though the time-course, polarity, and relative amplitudes of all estimates were acceptable.

Overall, tetrahedral and hexahedral (*shifted*) numerical estimates corresponded well with the analytical estimates (gray lines in panel Figure 13) in both waveform and relative amplitudes. The width, latency, and amplitude of population spikes were well-approximated across all transverse locations with the greatest errors being in the exposed infrapyramidal blade due to relative proximity to the stimulating location. A greater portion of error was also concentrated in the after-hyperpolarization phase of the response (+8.0–12.0 ms).

## Discussion

**Figure 14:**
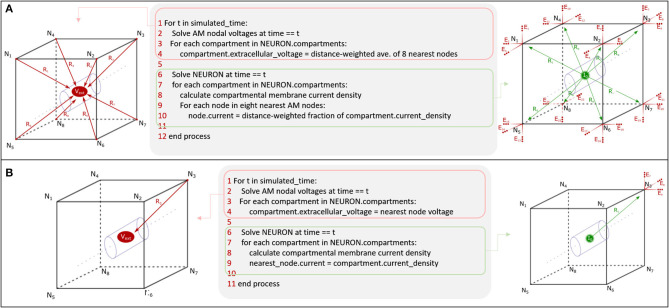
Synthesis of feedforward AM-NEURON methods demonstrated in Bingham et al. (2018a) and feedback AM-NEURON examined in this manuscript via current **(A)** splitting and **(B)** shifting yields an algorithm for bidirectional communication in mechanistic models of neural tissue systems and volume conduction models. The above pseudocode would be the same for tetrahedral geometry except for the substitution of the number of nodes to interpolate from (4 vs. 8) in **A**. Except in very coarse meshes, shifting (a simpler algorithm) produced results comparable to current splitting in hexahedral or tetrahedral models.

The goal of this study was to demonstrate the theoretical foundations of application of a numerical method to the estimation of LFPs given neural model-driven current sources which are broadly distributed in space and time. This work was focused specifically on field estimation problems arising from having endogeneous current sources which do not spatially align with volume conductor meshed nodes and, thus, must be split, merged, or shifted in space. Having such a method enables the implementation of critical features of tissue for both feedforward and feedback tissue-electrode and tissue-tissue interactions; these features include tissue dielectric heterogeneity and anisotropy. The ability to accurately estimate (i) electric fields generated in neural tissue by stimulating electrodes, (ii) evoked behavior of neural tissue that lies within that volume, and (iii) the electric fields that are generated by that behavior completes the critical components of an algorithm that couples feedforward and feedback interactions between virtual electrical stimulating, recording devices, and computational models of neural tissue. An algorithm for bidirectional communication between AM and NEURON domains could prove a powerful approach to understanding electrode-based brain-machine-interfaces and electroceutical devices. While the accuracy of the numerical method with respect to analytical solutions could have been performed with randomized non-biological current sources, previous work enabled the use of a highly detailed *in vitro* dentate slice rat model, and the LFP solution reflects the behavior of this model (Bingham et al., 2018a). Accurate use of the Admittance Method for LFP estimation that corresponds to *in vivo* behavior would have required modifications of the underlying NEURON model to capture the differences between *in vitro* and *in vivo* hippocampal tissue including differing dielectric properties, tissue geometry, network topology, etc. Therefore, effective use of this algorithm for neuroscience applications requires that modelers first construct a meaningful mechanistic model of the neural tissue they wish to study. The general value of this algorithm for neuroscience applications is limited by the realism of the interfacing models: (i) volume conduction model design is complicated by geometric and anatomic variety of electrodes and tissue and (ii) prediction of neural tissue system behavior is complicated by the nuances of tuning biologically detailed models of neurons and synapses. Through synthesis of the results presented in this paper and those that provided the feedforward AM-NEURON model described previously, we propose a bidirectional algorithm that deepens the cross-scale links between models of bulk-tissue dielectric behavior and cellular and network behavior of complex neural tissue systems (Figure 14).

A stable and efficient approach to solving fields arising in tissue from endogenous current sources allows us to begin inquiry into several new areas of work. Some of these include the following: (i) providing clarity regarding the extent to which volume conduction of transmembrane currents reinforces population synchrony, (ii) use of the model framework to validate and optimize spike-sorting algorithms for engineering applications, and (iii) investigation of more fundamental questions about the biology of specific neural circuits by determining with greater accuracy which components of a neuron or neurons in a network actually cause the features that emerge in evoked potentials (Einevoll et al., 2012). Each of these questions represents an avenue of work enabled and encouraged by the modeling framework presented herein.

## Limitations and Alternatives

While models constructed via this proposed algorithm may help investigators answer many questions related to brain-computer-interfaces and their useful application, it is not without limitations.

We have demonstrated and quantitatively evaluated an approach to estimating electric fields that arise from endogenous current sources. This is a general method that can be applied to the study of any neural tissue system with a few important caveats. As our study system, we adopted an AM-NEURON model of the rat hippocampus; many features of this tissue system proved critical for accurate prediction of spatiotemporal patterns of activity (Buzsáki et al., 1979). It is likewise critical to capture the principle geometry and topology of neural networks and dielectric properties of various regions of the tissue systems being modeled (Tveito et al., 2017). When combined, these tissue specific properties sculpt the geometry of electric fields generated by neuronal activity. Further, tissue, behavioral, and environmental variables can change AM meshing requirements and model parameters. This constitutes a substantial hurdle for proper implementation of AM for LFP estimation; the need for mesh generation and refinement is a limitation of this modeling approach, though not one that disadvantages AM relative to alternative numerical methods that face similar meshing challenges (e.g., FEM) (Pridmore et al., 1981).

### Spatial Resolution of Sources and Meshes

One feature of model performance that drew our attention and deserves comment is the potential for mechanistic differences in error in the evoked potential phase (+3.0–12.0 ms) and those from the artifact phase (+0–2.0 ms). While beyond the scope of this present report, these differences warrant further analysis and may become the focus of subsequent studies. Because recording locations are often very far from the stimulation location, artifacts in LFPs far from the stimulation can be estimated accurately with very coarse meshes without adequately capturing the activity nearest to the virtual recording electrode (Nowak and Bullier, 1996). Minimizing error in both population spike phase and artifact phase for any given location potentially requires two meshes of differing resolutions or meshes with two resolutions, near and distal to recording sites of interest in addition to the stimulating location. Because the error types appear to be separable in time (artifact and EP phases), a different mesh could be used to optimize prediction performance within each time span.

### Floating Point Arithmetic

When implementing very large numerical problems, one known source of error is floating point arithmetic. As loop currents are calculated within the spatial circuit and the voltage matrix is solved, values are inevitably rounded to a reasonable precision. As a result, small errors can accumulate. Limitations in arithmetical accuracy are imposed by floating-point precision capabilities of both computer hardware and programming languages. By default, Python uses 53 bits of precision and this was carried through all operations in construction of our RC circuits and solutions thereof. While floating point errors are academically interesting and their analysis is critical for many multiphysics simulation problems, this source of error (at 53 bits of precision) is likely to be negligible with respect to other sources of error in computational neural systems and modeling (e.g., mesh resolution, geometry, neuronal biophysics, etc.).

### Mesh Quality

Mesh design challenges represent one specific limitation for most numerical approaches to solving electromagnetic fields, the AM included. For every new problem and new tissue geometry, new meshes must be designed as there is not likely to be a universally optimal mesh volume, fundamental geometry, or resolution. Technically speaking, each model must find a new optimum for the following three variables: geometry, resolution, and mesh material properties (e.g., admittance). For LFP estimation, these calculations must also consider the spatiotemporal distribution of endogenous current sources.

### Solvers and Model Complexity

Computational demands and model complexity represent additional critical challenges that must be faced. Approaches to mitigating these demands are being developed by the authors and others who have a need for mature electromagnetics numerical solvers. Potential approaches include mesh reduction or refinement and parallel solution of the AM. Alternative solvers may be considered which have already implemented massively parallel solution of RC circuits, such as the Xyce environment distributed by Sandia National Labs (Hutchinson et al., 2002). Should there be no such optimization of FEM-style methods, then compute-time advantages of the numerical method over analytical methods could potentially be negated by dramatic parallelization of integral-based solutions.

Interestingly, for LFP estimation problems it is quite likely that mesh/circuit solution is not the only computational bottleneck. Preprocessing is not a trivial step in the process of numerical solution of LFPs. Because this study involved so many simulations of varying resolution, it required repeated discretization of current sources into their respective mesh model elements. For very small problems preprocessing is trivial; but computing the pairwise relational distance of millions of points is very time-consuming and memory intensive. Our approach to alleviating this challenge was to massively parallelize the solution, but an ideal tool that maximizes impact in the neural computing community would not require use of a high-performance computing (HPC) cluster. While the authors had access to HPC resources, the implementation to be distributed only requires a single-machine and will be multi-thread enabled. This means that investigators must be especially aware of the potential performance impact of increased quantities and spatial distribution of current sources relative to their meshed models. Future implementations may potentially be capable of performing preprocessing and/or solution via GPU, as many of the respective operations are reduceable to matrix algebra first principles.

Ultimately, relative compute-time performance of analytical vs. numerical methods is dependent upon compute-hardware, the specific software implementation, and the spatial resolution of both current source inputs and recording locations to be solved. Thus, while there remain significant advantages for numerical over analytical methods in solution of complex and heterogeneous problems, it is difficult to conclude that numerical methods are always faster or more efficient than analytical for homogeneous volume conduction problems. However, for inhomogeneous problems, there is significant advantage afforded by application of a numerical method over analytical.

## Conclusions

The present study demonstrates an intuitive numerical approach to estimating local field potentials generated by a detailed computational model of cortical tissue. AM-NEURON LFP estimation, the analysis presented herein, and its synthesis with earlier work makes three recognizable contributions to model-based analysis of complex neural systems and electroceutical devices: (i) validation of a fundamental approach to incorporating incident and dynamic current sources into a pre-existing meshed model, (ii) suggestion of an equivalent-circuit algorithm that unifies intracellular and extracellular neuronal models, and (iii) clarification of the impact of mesh geometry on computational performance and model accuracy. Together, these contributions point the way forward in the development of more sophisticated models that seek to understand the bi-directional interaction between brains and brain-computer interfaces or electroceutical devices.

## Data Availability Statement

Project code to generate and solve fields arising from endogenous current sources can be found in a Python library (Neurospice) that is publicly distributed on Github (https://github.com/bingsome/Neurospice).

## Author's Summary

Rapid advances in computing and neural interfacing technologies have encouraged the development of model-based approaches to predicting brain behavior. Combining meshed models with discrete solutions to the partial differential equations which govern the spread of currents forms the foundation for powerful models of electrical stimulation of neural tissue. But, as these models increase in complexity, the need becomes more severe for the development of sophisticated computational approaches to understanding the fields generated by not only active electrodes but also active neurons. Due to the spatial, temporal, and magnitudinal differences in electrode vs. neuron generated fields, different numerical algorithms may be required to accurately predict voltage gradients throughout affected volumes when generated by numerous neuronal sources. To address this concern, we have conducted a study which explores the impact on field estimation of mesh geometry and algorithms for handling spatial mismatches between current sources and meshed nodes. Establishment of a robust algorithm for numerical estimation of field potentials arising from neural activation could provide a path toward more predictive modeling of large-scale recording systems, neural network behavior, and extracellular neuron-neuron interactions.

## Author Contributions

CB constructed the models and conducted the analysis. JP and CG contributed to mathematical formulations. J-MB, JP, and CG contributed to project and manuscript design. J-MB, DS, GL, and TB oversaw development of models and analysis. GL and TB were involved in project conception and procurement of funding. All authors contributed to the article and approved the submitted version.

## Conflict of Interest

The authors declare that the research was conducted in the absence of any commercial or financial relationships that could be construed as a potential conflict of interest.
