# Supplementary material for: Admittance Method for Estimating Local Field Potentials Generated in a Multi-Scale Neuron Model of the Hippocampus
Source: Front Comput Neurosci. 2020 Aug 4;14:72. doi: 10.3389/fncom.2020.00072 (PMC7417331; doi:10.3389/fncom.2020.00072)
Supplement: Supplementary file 1 [file Presentation_1.PPTX]

## Slide 1
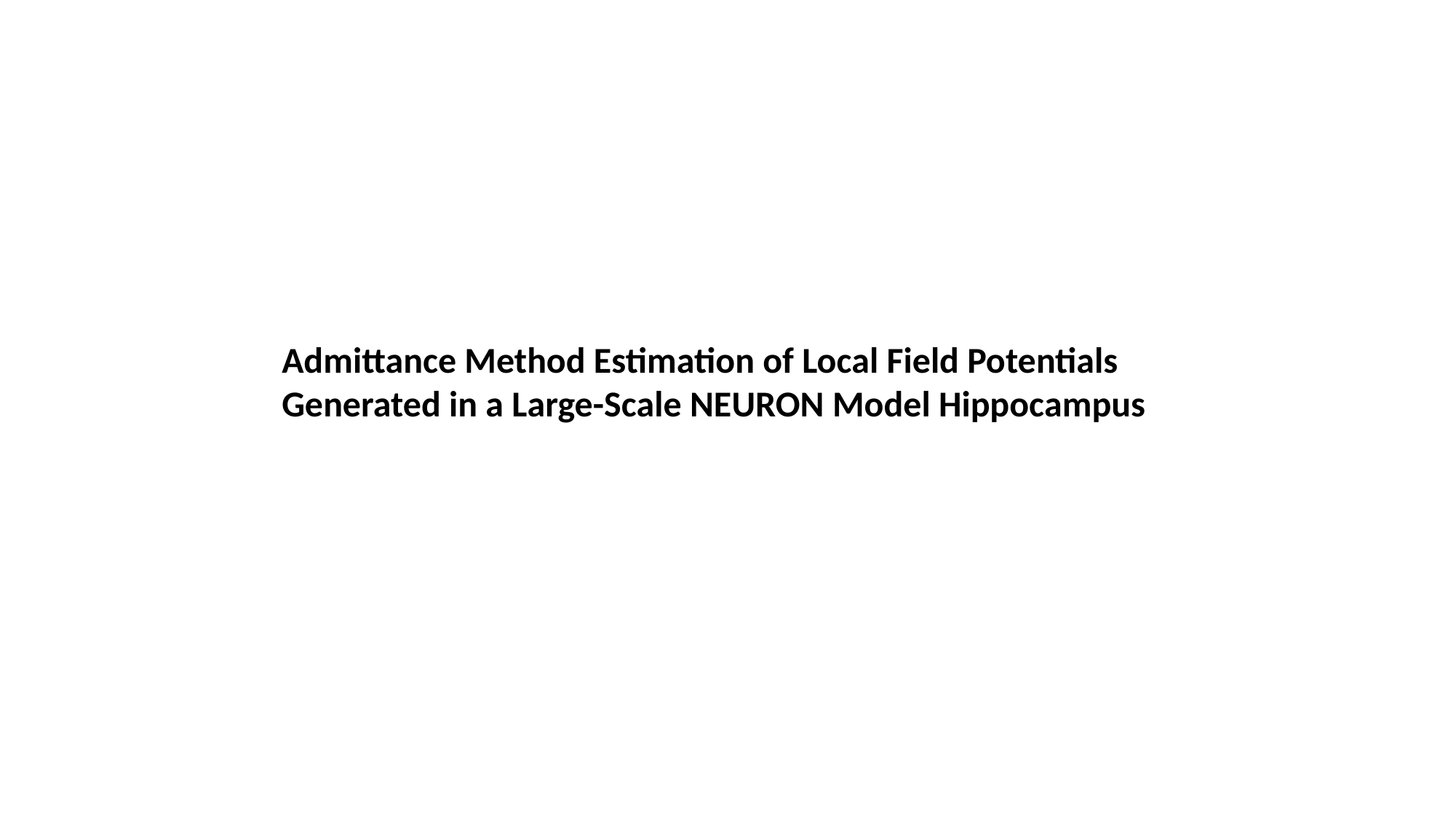

Admittance Method Estimation of Local Field Potentials Generated in a Large-Scale NEURON Model Hippocampus

## Slide 2
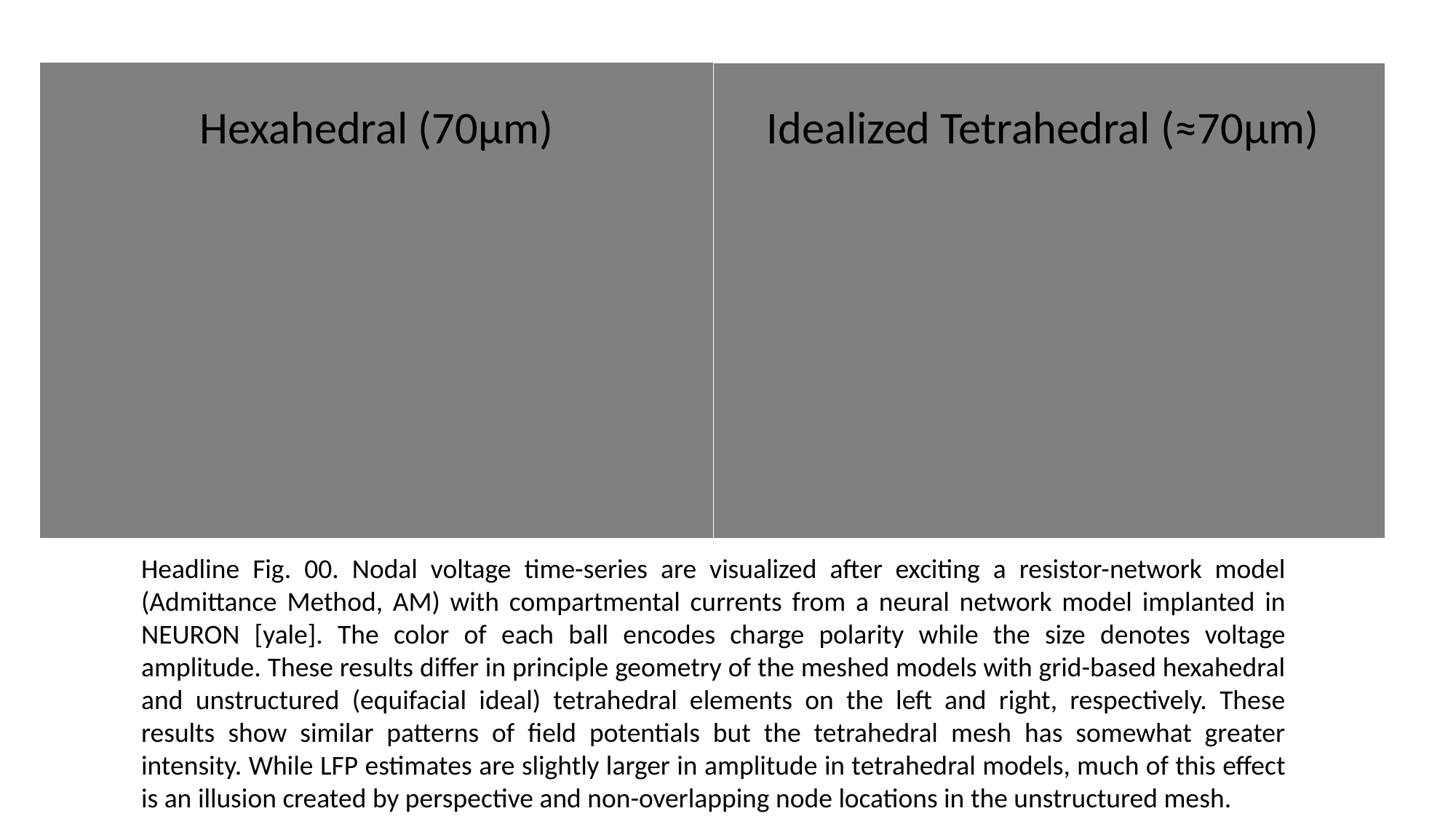

Hexahedral (70µm)
Idealized Tetrahedral (≈70µm)
Headline Fig. 00. Nodal voltage time-series are visualized after exciting a resistor-network model (Admittance Method, AM) with compartmental currents from a neural network model implanted in NEURON [yale]. The color of each ball encodes charge polarity while the size denotes voltage amplitude. These results differ in principle geometry of the meshed models with grid-based hexahedral and unstructured (equifacial ideal) tetrahedral elements on the left and right, respectively. These results show similar patterns of field potentials but the tetrahedral mesh has somewhat greater intensity. While LFP estimates are slightly larger in amplitude in tetrahedral models, much of this effect is an illusion created by perspective and non-overlapping node locations in the unstructured mesh.

## Slide 3
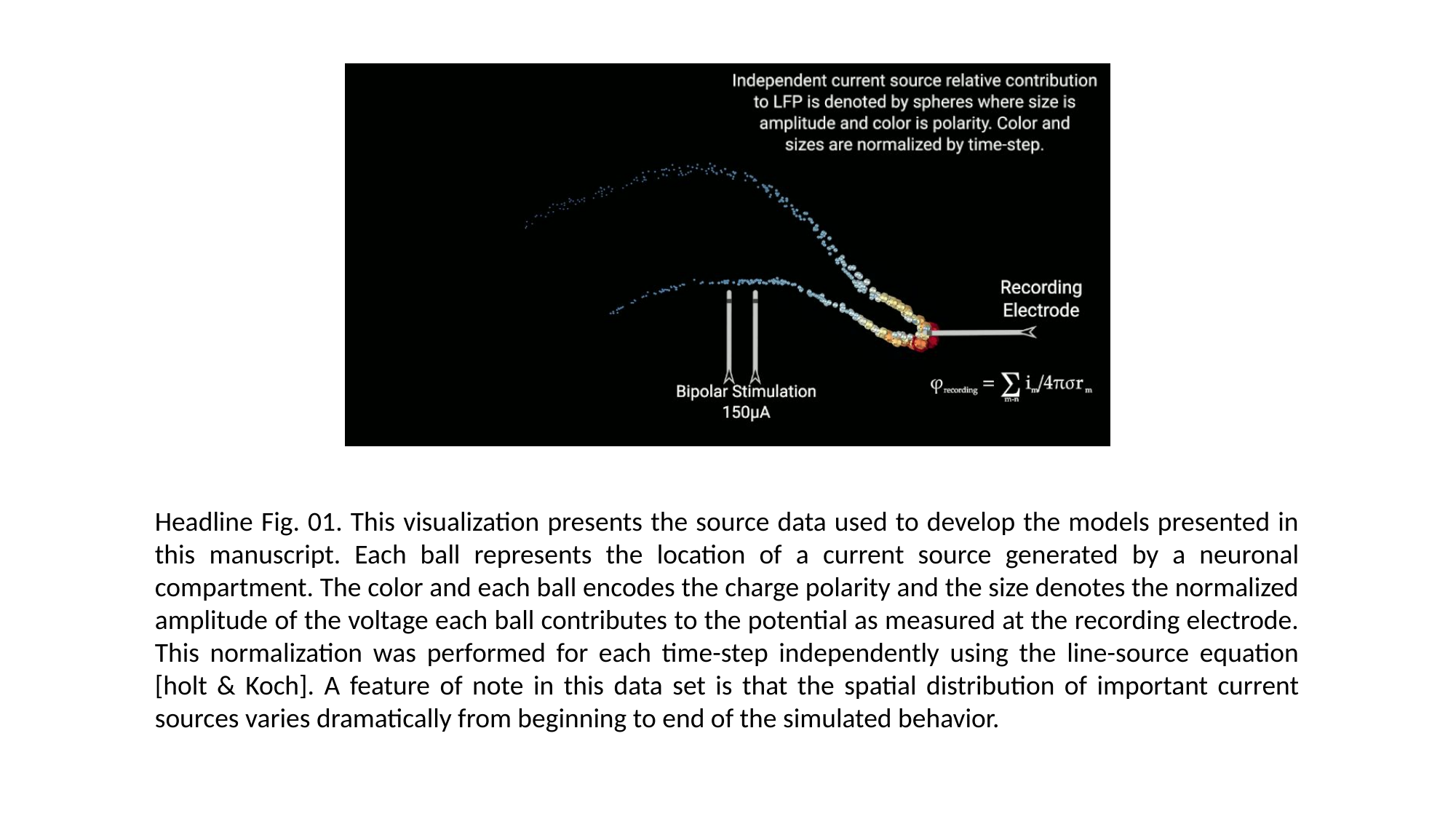

Headline Fig. 01. This visualization presents the source data used to develop the models presented in this manuscript. Each ball represents the location of a current source generated by a neuronal compartment. The color and each ball encodes the charge polarity and the size denotes the normalized amplitude of the voltage each ball contributes to the potential as measured at the recording electrode. This normalization was performed for each time-step independently using the line-source equation [holt & Koch]. A feature of note in this data set is that the spatial distribution of important current sources varies dramatically from beginning to end of the simulated behavior.
